# On the Optimal Size and Composition of Customs Unions: An Evolutionary Approach

**DOI:** 10.1007/s10614-022-10307-w

**Published:** 2022-08-17

**Authors:** Takfarinas Saber, Dominik Naeher, Philippe De Lombaerde

**Affiliations:** 1 Lero – The Irish Software Research Centre, School of Computer Science, National University of Ireland Galway, Galway, Ireland; 2 Department of Development Economics, University of Goettingen, Goettingen, Germany; 3 Neoma Business School, Rouen, France; 4 United Nations University Institute on Comparative Regional Integration Studies (UNU-CRIS), Brugge, Belgium

**Keywords:** Regional integration, Customs unions, Multi-objective optimisation, Evolutionary algorithm, C6, F13, F15, F60

## Abstract

Customs unions enable countries to freely access each other’s markets, which is thought to increase intra-regional trade and economic growth. However, accession to a customs union also comes with the condition that all members need to consent to a common external trade policy. Especially if countries feature different economic structures, this may act as a force against the creation of large customs unions. In this paper, we propose a new mathematical approach to model the optimal size and composition of customs unions in the form of a bi-objective combinatorial non-linear problem. We also use a multi-objective evolutionary algorithm (NSGA-II) to search for the best (non-dominated) configurations using data on the trade flows and economic characteristics of 200 countries. Our algorithm identifies 445 different configurations that are strictly preferable, from a global perspective, to the real-world landscape of customs unions. However, many of these non-dominated configurations have the feature that they improve outcomes for the world as a whole, on average, but not for all individual countries. The best configurations tend to favour the creation of a few large customs unions and several smaller ones.

## Introduction

If one focused only on economic considerations, what would be the optimal number and composition of customs unions (CU) in the world? The primary purpose of a CU is to make it easier for the member countries to trade freely with each other. The underlying idea is that trade (as long as it is based on mutual consent) makes all involved parties better off. Reducing obstacles to trade between any two countries, e.g., by establishing a customs union, can therefore be seen as a desired outcome (Baldwin & Venables, 1995; Henrekson et al., 1997; Fernández & Portes, 1998; Te Velde, 2011). At the same time, it has been shown that CU members can benefit from a terms-of-trade effect in their trade relations with third countries (Krugman, 1991; Olarreaga et al., 1999).

However, there are also costs involved as a CU grows larger. One important element of these costs is based on increasing dissimilarity. Analogously to the Kenen criterion (Kenen, 1969) in Optimum Currency Area (OCA) theory, the optimal composition of a CU is such that its members feature similar economic structures, which makes it easier for them to agree on a common market for their goods and a common strategy regarding trade policy with nonmembers.^1^ As a CU grows larger, the economic structures of its members tend to become more diverse, e.g., with respect to the sectoral composition of their GDP. Hence, there exists a trade-off: a larger CU is desirable because it enhances the usefulness for its members of being part of a common market with fewer trade barriers, but it also has drawbacks in terms of increased dissimilarity. The optimal CU landscape trades off the benefits associated with each CU against the respective costs.

A crucial difference between CUs and free trade areas (FTAs) should be pointed out in this respect. The dissimilarity-trade nexus is indeed rather ambiguous when trade liberalization is considered more in general. As is well known from international trade theory, both dissimilarity and similarity can lead to more trade, but to two different types of trade: inter-industry and intra-industry trade, respectively. Both lead to gains-from-trade. However, as the CU implies a common external trade policy, or at least a common external tariff (CET), a CU does not seem to be necessarily compatible with a dissimilarity scenario (leading to trade creation of the inter-industry type). Real-world experiences with CUs seem to corroborate this expectation. Most CU projects in the world (see Table 1) have not been completed and are in practise only partial CUs with exceptions and perforations. The political economy of CUs is complex and it is often difficult to agree on a CET which covers 100 percent of the tariff universe (Olarreaga & Soloaga, 1998; Laens & Terra, 2005; Borchert & Magntorn, 2018; Vilela, 2020). Five of the listed CUs have been signed under the enabling clause.^2^ Incompleteness of a CU reduces its functioning considerably because it does not allow to move towards the abolition of intra-regional border controls (in combination with rules of origin), which is in principle a key characteristic of a CU thanks to its harmonized CET and complementary measures to integrate markets.

The relatively simple logic described above motivates our analysis. Specifically, we simulate the optimal size and composition of CUs in the world based on two empirically quantifiable indicators: (i) the intra-regional trade share as a proxy measure of the benefits associated with a given CU, and (ii) a dissimilarity score capturing differences in the sectoral composition of member countries’ GDP that constitute a barrier for the creation of large CUs. As will be clear from the definition of the two indicators given below, both tend to increase with the size of the CU (i.e. with the number of member countries) and will reach their maximum values when the CU coincides with the world. Or, to put it differently, the intra-regional trade share will tend to increase with the CU size, while similarity will decrease. However, whereas the dissimilarity score will monotonically increase with the size of the CU following a random expansion path, the intra-regional trade share will increase non-monotonically and will depend on the trade patterns of the sequential accession states.

In general, identifying the single best solution to the described trade-off would require us to make assumptions about the relative roles of the two involved factors, e.g., by specifying a welfare (utility) function based on the two factors. To the best of our knowledge, these assumptions would have to be *ad hoc* as there is no theory or empirical evidence available that could guide these assumptions. We therefore follow a different approach here, which is to characterize different possible CU configurations (based on the world’s current set of countries and borders) as either dominated or non-dominated by alternative solutions. A dominated solution is defined as a feasible CU configuration for which there exists another solution that features both a higher integration score and a lower or equal dissimilarity score (or a lower dissimilarity score and a higher or equal integration score). If no such superior solution exists, then the CU configuration is a non-dominated solution.

Since it would be impractical to evaluate all possible CU configurations, we make use of a multi-objective evolutionary algorithm adapted from bio-inspired optimisation literature, i.e., the Non-dominated Sorting Genetic Algorithm-II [NSGA-II (Deb et al., 2002)], to derive non-dominated solutions to this problem. The set of all non-dominated solutions form what is called a Pareto front: in this set, it is impossible to find any solution better in all objectives than any of the other solution in the set. NSGA-II is a successful multi-objective metaheuristic that is widely used in many real-world applications (e.g., Saber et al., 2018, 2020).

We then compare the non-dominated solutions to the real-world CU landscape (in the period 2014–2018) in three different ways: (i) from a global perspective where we analyse the average integration score and the average dissimilarity score across all countries, (ii) from a customs union perspective where we study the integration score, dissimilarity score, and sizes of the CUs composing every non-dominated solution, and (iii) from a country perspective where we evaluate the gain/loss to each country when moving from the CU landscape in the real world to the identified non-dominated solutions.

The obtained insights contribute mainly to the economic literature on regional integration [see the surveys in Baldwin and Venables (1995), Te Velde (2011)]. In recent years, there has also been an increasing interest in empirical measures of regional integration outcomes by policymakers and international institutions such as the United Nations, World Bank, and regional development banks (AfDB, 2016; Naeher, 2015; Naeher & Narayanan, 2020; UNESCAP, 2020). The paper most closely linked to our analysis is perhaps the study (De Lombaerde et al., 2021) which uses a machine learning approach based on a network clustering algorithm to evaluate the composition of real-world CUs. However, this study only considers a single outcome (regional integration shares) and does not attempt to solve for an optimal solution of the composition of CUs as we do below. Our analysis thus contributes to the existing literature both by proposing a new method to empirically evaluate the composition of CUs and by providing deeper insights that also take into account the cost associated with forming a CU.

The rest of the paper is organized as follows. Section 2 provides a formal definition of the problem we solve. Section 3 describes the multi-objective approach as well as the experimental setup. Section 4 reports and analyses our experimental results. Section 5 concludes.

## Problem Definition

In this section, we define the concept of a region (i.e., CU and provide a mathematical formulation for the regional integration problem as a bi-objective combinatorial problem.

### Regional Integration

Consider a set of countries ${\mathcal {C}}=\{c_1,c_2,\ldots ,c_n\}$ and their set of borders ${\mathcal {B}}$, with $b_{i,j}\in {\mathcal {B}}$ being the border for any two bordering countries $c_i$ and $c_j$ in ${\mathcal {C}}^2$. We follow the definition of a region adopted in De Lombaerde et al. (2021).

#### Definition 1

A region *r* is defined as a set of transitively bordering countries. Any two countries $c_i$ and $c_j$ within the same region *r* are transitively bordering each other if there exists a sequence of borders $(b_{i,w}, b_{w,x}, \ldots , b_{y,z}, b_{z,j})$ such that $\forall b\in \{b_{i,w}, b_{w,x}, \ldots , b_{y,z}, b_{z,j}\}~b \in {\mathcal {B}}$, and $\forall c \in \{c_w, c_x, \ldots , c_y, c_z\}~c$ is also in the region *r*.

### Mathematical Model

We are interested in identifying regional configurations; that is, assigning each country to exactly one region. There are various ways to mathematically model this problem, for example as a grouping of countries, partitioning of countries, or selection of borders. Our modeling approach is based on the latter formulation: selection of borders whereby all countries connected by the selected borders form a region. Our choice for this formulation ensures that all solutions are feasible. However, it also means that several solutions are symmetrical (i.e., solutions with different representations, but that have the same composition). Our proposed model is combinatorial. Its constraints are linear. However, its objectives are not. Therefore, this limits us from using Mixed-Integer Linear Programming (MILP (Saber et al., 2015)) solvers or multi-objective exact optimisation approaches that leverage them (e.g., epsilon constraints (Saber et al., 2017)).

#### Border Selection

We define the binary variables $x_{i,j}$ for every $(c_i,c_j) \in {\mathcal {C}}^2$ which indicate whether a border between $c_i$ and $c_j$ exists and has been selected for the region:

1 $$\begin{aligned} b_{i,j}\in {\mathcal {B}} \implies x_{i,j} \in \{0,1\} \end{aligned}$$

2 $$\begin{aligned} b_{i,j}\notin {\mathcal {B}} \implies x_{i,j} = 0 \end{aligned}$$

For border symmetry purposes:

3 $$\begin{aligned} (c_i,c_j) \in {\mathcal {C}}^2,~~~ x_{i,j} = x_{j,i} \end{aligned}$$

Therefore, the number of decision variables in our model is limited to $|{\mathcal {B}}|/2$ binary variables, i.e., the number of borders without the symmetry.^3^

##### Definition 2

A solution *S* to the regional configuration problem is a mapping of all binary variables $x_{i,j}$ to 0 or 1, such that $b_{i,j}\in {\mathcal {B}}$ and $i<j$.

#### Created Regions

Knowing that there are $|{\mathcal {C}}|$ countries, there is a maximum of $R= |{\mathcal {C}}|$ simultaneously existing regions (i.e., when each country forms its own region). Therefore, let the binary variables $y_{i}^{r}$ ($|{\mathcal {C}}|\times |{\mathcal {C}}|$ in total) be such that for every country $c_i\in {\mathcal {C}}$ and every region $r \in \{1..R\}$, $y_{i}^{r}$ is set to 1 if $c_i$ is part of *r* and 0 otherwise.

Every country can only be part of one and only one region:

4 $$\begin{aligned} \forall c_i\in {\mathcal {C}}, ~~~~~ \sum _{r\in \{1..R\}} y_{i}^{r}~=~1 \end{aligned}$$

Note that symmetrical solutions can be reduced by restricting equation (4) and forcing an order on what region each country can belong (i.e., each country is either in an earlier region, or is in a new region starting with itself):

5 $$\begin{aligned} \forall c_i\in {\mathcal {C}}, ~~~~~ \sum _{r\in \{1..i\}} y_{i}^{r}~=~1 ~~\text {and}~~ \sum _{r\in \{i+1..R\}} y_{i}^{r}~=~0 \end{aligned}$$

#### Connected Regions

Given a selection of borders *S*, every two countries within the same region must border each other, or transitively border each other through countries within the same region. To enforce the connectivity of all countries within the same region based on the selected borders, we use the constraint below.

6 $$\begin{aligned} ~\forall ~(c_j, c_j)~\in {\mathcal {C}}^2, ~\forall ~r\in \{1..R\},~~~ x_{i,j} = 1 \implies y_{i}^{r} = y_{j}^{r} \end{aligned}$$

### Mean Regional Integration Score

Every region *r* is associated with a regional integration score *RI*(*r*) which is a quantitative measure of the intra-regional trade share given by

7 $$\begin{aligned} RI(r) = \frac{ \sum _{(c_i,c_j)\in {\mathcal {C}}^2 ~|~ y_{i}^{r}=1,~ y_{j}^{r}=1} T(c_i,c_j) }{ \sum _{c_i \in {\mathcal {C}} |y_{i}^{r}=1} \sum _{c_j\in {\mathcal {C}}} T(c_i,c_j) } , \end{aligned}$$

where $T(c_i,c_j)$ is the sum of trade exports and imports between countries $c_i$ and $c_j$.

Given that the purpose of a CU is to facilitate and increase cross-border trade flows between its member states, we seek to find solutions that maximise the integration scores given by equation (7). Specifically, we set up our algorithm so as to minimise the additive inverse (i.e., multiplied by -1) of the mean integration score over all regions $r \in \{1..R\}$ that contain at least one country (i.e., $\sum _{c_i\in {\mathcal {C}}} y_i^r ~>~0$):

8 $$\begin{aligned} Minimise:~~ -~~ \frac{ \sum _{r\in \{1..R\} ~|~ \sum _{c_i\in {\mathcal {C}}},~ y_i^r>0} RI(r) }{ |\left\{ r~~|~~ r\in \{1..R\}, ~\exists c_i\in {\mathcal {C}}, ~y_i^r>0 \right\} |} \end{aligned}$$

### Mean Economic Dissimilarity Score

Every country $c_i\in {\mathcal {C}}$ has a particular value of GDP. Moreover, countries differ in their economic structure, such as the share of GDP generated in different economic sectors $s \in {\mathcal {E}} = \{\text {Industry, Agriculture, Manufacturing, Service}\}$. Analogously to the Kenen criterion in OCA theory discussed in Sect. 1, increasing differences in economic structure constitute an obstacle to larger sized CUs, because it makes it more difficult for the members to agree on a common trade policy. In our analysis, this is captured by considering differences in the value added of each sector as a percentage of each country’s total GDP. Therefore, every country $c_i\in {\mathcal {C}}$ has a GDP proportion $p(c_i, s)$ for every sector $s \in {\mathcal {E}}$.

Given the diversity in economic structures of countries within the same geographic region, every region *r* is associated with an overall economic dissimilarity score *D*(*r*, *s*) for each economic sector $s \in {\mathcal {E}}$:

9 $$\begin{aligned} D(r,s) = \max _{c_i \in {\mathcal {C}} |y_{i}^{r}=1} p(c_i, s) - \min _{c_i \in {\mathcal {C}} |y_{i}^{r}=1} p(c_i, s) \end{aligned}$$

An overall economic dissimilarity score *D*(*r*) for a particular region *r* is the average of its sectoral dissimilarity scores *D*(*u*, *r*).

10 $$\begin{aligned} D(r) = \frac{ \sum _{s\in {\mathcal {E}}} D(r,s) }{ |{\mathcal {E}}|} \end{aligned}$$

As discussed above, economic dissimilarity represents a cost (or barrier) to the creation and expansion of CUs. We therefore seek to find solutions that minimise the dissimilarity scores given by equation (10). Specifically, we set up the algorithm so as to minimise the mean economic dissimilarity score over all regions $r \in \{1..R\}$ that contain at least one country (i.e., $\sum _{c_i\in {\mathcal {C}}} y_i^r ~>~0$):

11 $$\begin{aligned} Minimise:~~ \frac{ \sum _{r\in \{1..R\} ~|~ \sum _{c_i\in {\mathcal {C}}},~ y_i^r>0} D(r) }{ |\left\{ r ~~|~~ r\in \{1..R\}, ~ \exists c_i\in {\mathcal {C}},~ y_i^r>0 \right\} |} \end{aligned}$$

## Evaluation Setup

In this section, we describe our experimental design^4^ in three parts: (i) the dataset, (ii) the multi-objective algorithm, and (iii) the setup of our system with the values defined for the parameters of our algorithm.

### Data

**Fig. 1:**
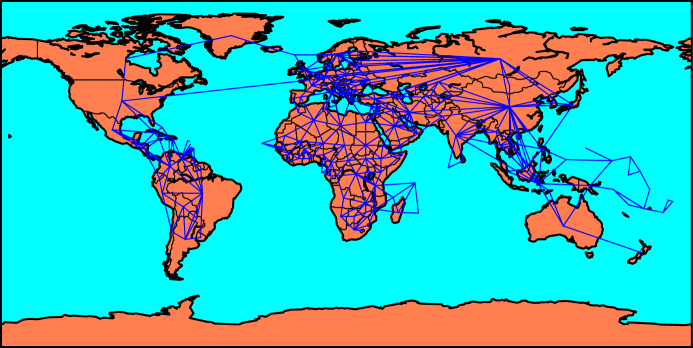
Land and maritime borders (in blue lines). (Color figure online)

All data used in this study are openly available from the following sources. The data on trade flows come from the Direction of Trade Statistics (DOTS) database of the International Monetary Fund (IMF). To limit the role of temporary fluctuations in trade flows and measurement error, we work with 5-year average values corresponding to the period 2014–2018. The data on the sectoral composition of GDP come from the World Bank’s World Development Indicator database. Our sample consists of 200 countries for which information on both land and maritime borders is available from GeoDataSource (2021). A complete list of the considered countries and borders is depicted by Fig. 1.

#### Real-World Custom Unions

We include in our analysis the real world CUs that are notified to the WTO and currently in force. A list of the included CUs and their composition is contained in Table 1. Note that this list differs slightly from the longer list of notified CUs in the WTO database for the following reasons:

- Our list does not include accessions separately as they collapse with the CU to which they refer.
- We do not include the EU-Andorra CU (entry into force: 01/07/1991) and the EU-San Marino CU (entry into force: 01/04/2002).
- COMESA is not included because, although its CU was formally notified to the WTO in 1995 under the enabling clause, it is still not operational (United Nations Economic Commission for Africa, 2021).
- The Russian Federation-Belarus-Kazakhstan CU (entry into force: 03/12/1997) is not included as it was absorbed by the EAEU.
- The West African Economic and Monetary Union (WAEMU) is not included as all its members are also part of ECOWAS.
- The EU-Turkey CU is not included as it is only a partial agreement, covering only industrial goods, and without a coherent common external tariff (De Lombaerde & Ulyanov, 2020).
- Also note that our analysis still refers to the EU-28. The UK was a Member State of the EU until January 2020, but the Withdrawal Agreement provided for a transition period during which the UK continued to be considered as a EU Member State for the purposes of relevant international agreements, including the customs union.
- Our analysis refers further to Mercosur-4. An accession protocol was signed between MERCOSUR member states and Venezuela in 2006, and the latter country became a full member in 2013. However, it was suspended in 2016.

In total, this gives us a set of 11 CUs.

**Table 1:** Real world customs unions

Customs Union (CU)	Countries
Andean Community (CAN)	Ecuador, Peru, Bolivia, Colombia
Caribbean Community and Common Market (CARICOM)	Saint Vincent and the Grenadines, Grenada, Trinidad and Tobago, Barbados, Guyana, Saint Lucia, Suriname, Antigua and Barbuda, Dominica, Saint Kitts and Nevis, Haiti, Jamaica, Bahamas, Belize
Central American Common Market (CACM)	El Salvador, Nicaragua, Costa Rica, Panama, Guatemala, Honduras
East African Community (EAC)	Burundi, Rwanda, Uganda, Kenya, Tanzania
European Community (EC)	Czech Republic, Slovak Republic, Austria, Hungary, France, Germany, Netherlands, Spain, Denmark, Estonia, Finland, Latvia, Lithuania, Sweden, Belgium, Bulgaria, Ireland, Luxembourg, Poland, Romania, UK, Croatia, Cyprus, Greece, Italy, Malta, Portugal, Slovenia
Economic and Monetary Community of Central Africa (CEMAC)	Cameroon, Equatorial Guinea, Congo, Gabon, Central African Republic, Chad
Economic Community of West African States (ECOWAS)	Guinea, Guinea-Bissau, Liberia, Sierra Leone, Cabo Verde, The Gambia, Senegal, Câte d’Ivoire, Ghana, Mali, Benin, Burkina Faso, Niger, Nigeria, Togo
Eurasian Economic Union (EAEU)	Kazakhstan, Russian Federation, Belarus, Armenia, Kyrgyz Republic
Gulf Cooperation Council (GCC)	Oman, United Arab Emirates, Qatar, Bahrain, Kuwait, Saudi Arabia
Southern African Customs Union (SACU)	Botswana, Namibia, Lesotho, South Africa, Eswatini
Southern Common Market (MERCOSUR)	Argentina, Uruguay, Brazil, Paraguay

### Algorithms

We define two algorithms: a random algorithm as a baseline, and a multi-objective evolution algorithm as our proposed approach.

#### Baseline: Random Search

Our work is the first to model and optimise the multi-objective regional integration problem. Therefore, there is no established state-of-the-art.

For our experiments, we define a random algorithm as the baseline against which we aim to improve. The baseline generates solutions with a random selection of borders (i.e., randomly assigns 0 or 1 to each border).

Since the random generation of solutions using a 0.5 probabibility for the selection of each border is likely to favour the creation of large CUs (Erdős & Rényi, 1960), we define 5 random searches with various probabilities for the selection of a border between 0.1 and 0.5:

- Random$_{\text {0.1}}$ with a 0.1 probability for selecting each border
- Random$_{\text {0.2}}$ with a 0.2 probability for selecting each border
- Random$_{\text {0.3}}$ with a 0.3 probability for selecting each border
- Random$_{\text {0.4}}$ with a 0.4 probability for selecting each border
- Random$_{\text {0.5}}$ with a 0.5 probability for selecting each border

#### Our Approach: Multi-Objective Evolutionary Algorithm

To optimise our multi-objective problem, we use the Non-dominated Sorting Genetic Algorithm-II (NSGA-II (Deb et al., 2002)); a successful multi-objective metaheuristic (particularly with 2 objectives) and widely used in many real-world applications (Saber et al., 2018, 2020).

This metaheuristic belongs to the genetic algorithm family, i.e., a population-based evolutionary process which matches individuals (i.e., solutions) of the population (i.e., a set of solutions) at each generation and mixes their features/characteristics (as the biological evolution would do with genes) to generate an offspring (i.e., children or newly generated solutions).

**Fig. 2:**
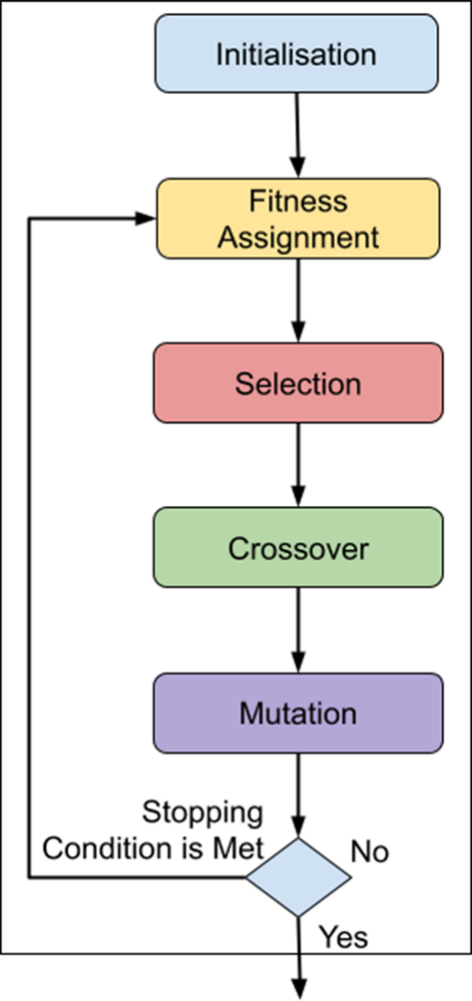
Main steps of genetic algorithms

Figure 2 shows an overview of the steps taken in genetic algorithms:

- *Initialisation*: generation of an initial population (a set of solutions). Often (and also in our case), the initial population is randomly generated.
- *Fitness Assignment*: computation of the objectives of each solution in the population (in our case, calculating the mean regional integration and the mean economic dissimilarity scores).
- *Selection*: selection of the ideal solutions to retain for the next generation of the genetic algorithm.
- *Crossover*: combines the features/characteristics of the solutions in the population to create new offspring solutions which combine their characteristics.
- *Mutation*: creates new offspring solutions by randomly changing the features/characteristics of the solutions in the population.

There exist several ways of doing crossovers, which cut the set of actual solutions into regular length segments and swap them with one another. In our case, we use the One-Point Cut Crossover between two parent solutions that generates two offspring solutions by swapping their border selection sequences at a randomly generated cut. We also use a Bit Flit mutation which has a certain probability of reversing the selection of every border in a solution (i.e., unselect the border if it is already selected, and select the border if it is not already selected).

After a generation has “passed” (i.e., the stopping condition is not met), some new solutions are kept (usually the fittest, those with the best objective values: low domination rank, but also some others that allow introducing some variety: high crowding-measure (Deb et al., 2002)), and others are suppressed. Hence the global population of solutions only improves (descendants worse than their parents are likely to be suppressed). Besides, the final generations tend to be well distributed over the front of non-dominated solutions.

NSGA-II allows to get a good dissemination of the solutions around the Pareto front^5^ and prevent their accumulation in some area of the search space. It also allows getting a smooth front and increases the number and the quality of the non-dominated solutions.

We use the implementation of NSGA-II that is provided in jMetalPy (implemented in python) and implement our problem/approach on top of it^6^.

these are the parameters for the NSGA-II used in our experiment:

- Population size: 1000
- Offspring population size: 1000
- Crossover: SPXCrossover with 0.8 probability
- Mutation: Bit-Flip with (1/#borders) mutation probability for each border selection

### Multi-objective Performance Metrics

To assess the performance of our algorithm against the baseline we use 5 well-known multi-objective performance metrics: 4 quality metrics (Hypervolume, Epsilon, Generation Distance, and Inverted Generation Distance) and 1 diversity metric (Spread).

- Hypervolume (HV, to maximise): computes the volume (measured in *k* dimensions of the problem’s search space) that is dominated by the Pareto front. In our case, we have two objectives, therefore, k is equal to 2.
- Epsilon ($\epsilon$, to minimise): evaluates the smallest distance that is needed for every solution in Pareto front to dominate the Reference front^7^.
- Generation Distance (GD, to minimise): evaluates the smallest distance needed for every solution in Pareto front to dominate the Reference front.
- Inverted Generation Distance (IGD, to minimise): evaluates average distance between every solution in Reference front and its closest solution in Pareto front.
- Spread (S, to maximise): computes the solutions’ distribution to evaluate their extent spread in Pareto front.

Due to the absence of the list of all *optimal* non-dominated solutions, we use an aggregate of all the solutions found by our algorithms over the different runs as the reference front.

## Results

In this section, we report and analyse results of our experimental evaluation.

### Performance Metrics

To evaluate the efficiency of our proposed approach based on NSGA-II, we would like to assess its achieved performance over generations in comparison to the baseline (i.e., random search with the different border selection probabilities).

**Fig. 3:**
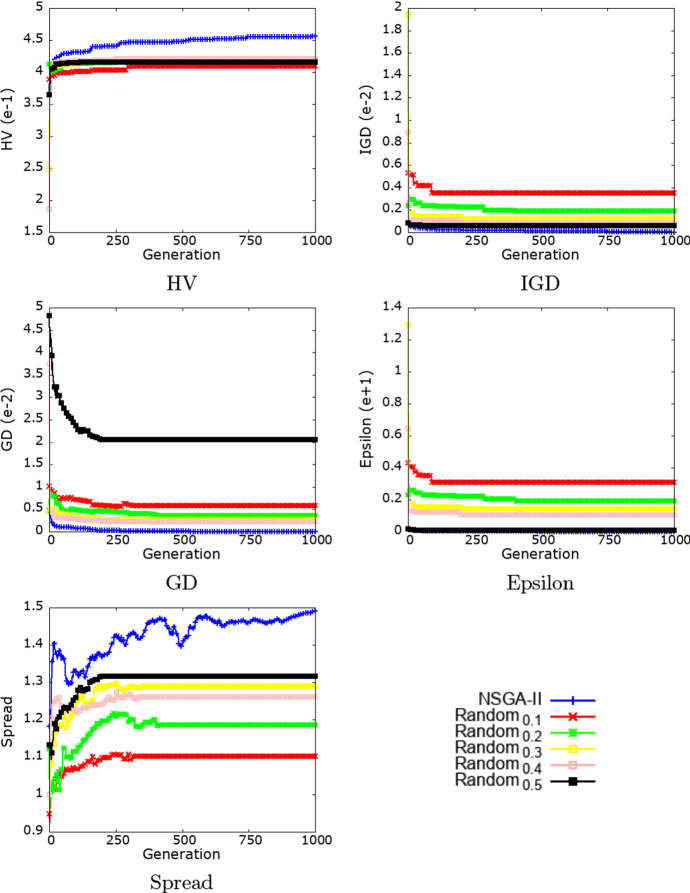
Evolution of the multi-objective performance metrics of both NSGA-II and the random baseline (with a probability of border selection between 0.1 and 0.5) over generations (result are averaged over 30 runs)

Figure 3 shows the evolution of the 5 multi-objective performance metrics over the different generations for each of the algorithms (results are averaged over 30 runs). Note that, while the random baselines have no notion of generation, every 1000 solutions are grouped in a generation.

Figure 3 indicates that NSGA-II achieves a better performance than the baseline (i.e., random search with all the different border selection probabilities) over all the used performance metrics.

We see that NSGA-II continuously improves the performance in terms of HV, IGD, GD and Epsilon over generations, which clearly shows that the evolutionary process is successful at finding better solutions over time. On the other hand, the improvement in Spread is not as regular as with other metrics: Spread increases overall between the start and the end of the algorithm, but goes through various phases of increases and decreases. However, this is not an issue as an algorithm can find better solutions, but close to each other.

We also see that the baseline search algorithms are also successful at improving all performance metrics. This is particularly facilitated by the fact that all solutions are feasible by design. It is also facilitated by the ease of covering several areas of the search space by only selecting fewer or more borders for each solution. However, following a significant performance increase in the first third of the generations, the performance of the random algorithms stagnates. Overall, we notice that larger border selection probabilities (i.e., 0.5 and 0.4) tend to yield a better performance on all metrics (except with GD where the probability 0.5 achieved worse results).

### Analysis of Non-dominated Solutions

We analyse the non-dominated solutions found by our evolutionary algorithm from three perspectives: global, customs unions, and country perspectives.

#### Global Perspective

**Fig. 4:**
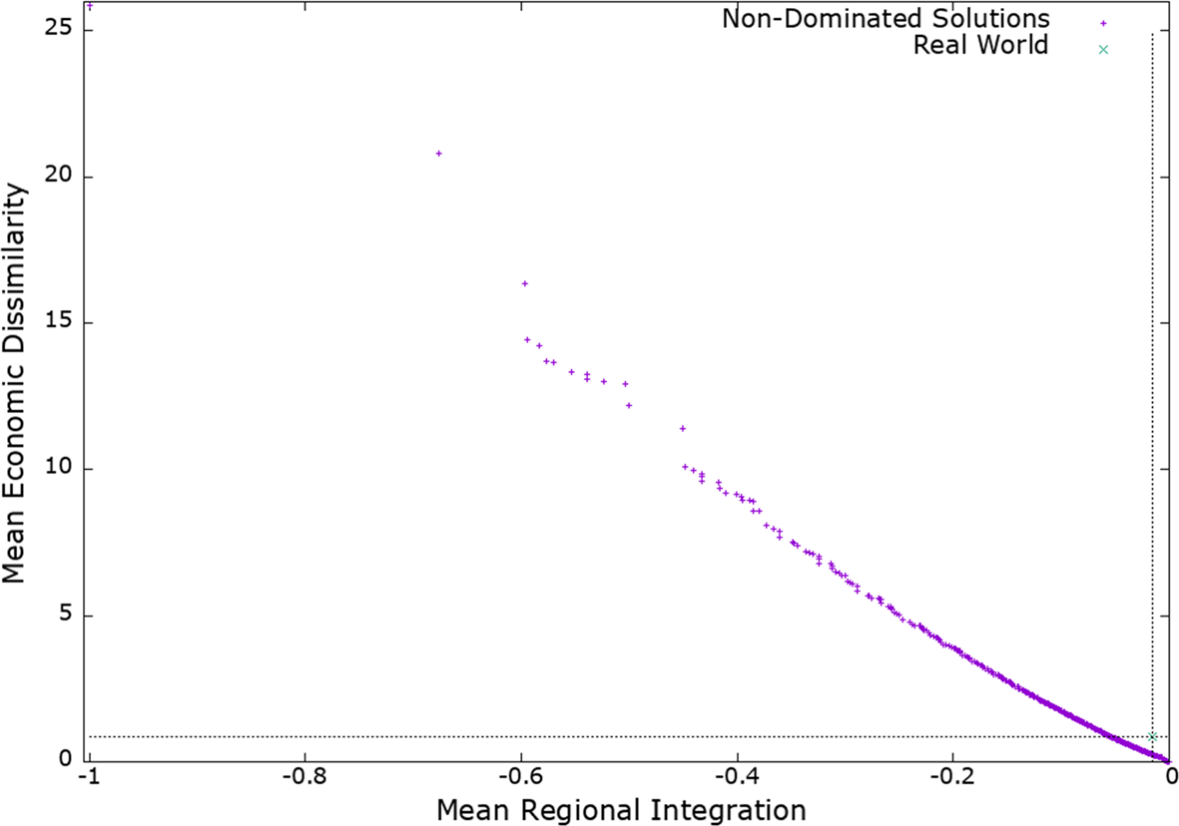
Non-dominated solutions found by NSGA-II (in purple) and configuration of real-world customs unions (in green cross). The two dashed reference lines (vertical and horizontal) delimit solutions that dominate the configuration of real world customs unions (i.e., solutions that perform better, on average, on both objectives). (Color figure online)

Figure 4 shows the achieved objectives (average integration score and economic dissimilarity score) of each non-dominated solution (regional configuration, in purple) found by the NSGA-II from the run that achieved the median HV. It also shows the average integration score and economic dissimilarity of the regional configuration that is composed of the real world CUs (in green cross).

There is a large number (1025) of non-dominated solutions. Many of these solutions do not belong to the convex region and are therefore impossible to find using a solver that optimises a weighted sum of both objectives (Laumanns et al., 2006). Moreover, since we are in a bi-objective context, each solution in Fig. 4 has an average integration score lower than the solutions on its right (i.e., solutions are sorted from left to right with an increasing average regional integration). Similarly, each solution in Fig. 4 has an average economic dissimilarity score lower than the solutions above it (i.e., solutions are sorted from bottom to top with an increasing average economic dissimilarity score.

We also see from Fig. 4 that non-dominated solutions are more densely concentrated near to the origin of the two objective scores (in the down right corner of Fig. 4). This is to be expected as large (absolute) integration scores tend to correspond to regional configurations with large CUs. For example, an integration score of -1 (see the solution in the upper left corner in Fig. 4) corresponds to the regional configuration with one CU comprising all countries in the sample. In contrast, low integration scores tend to correspond to configurations with small CUs. The smaller the size of the CUs, the larger is the number of potential regional configurations, and vice versa.

More importantly, we notice that a fair number of the non-dominated solutions are dominating the regional configuration composed of the real-world CUs. These solutions are shown in Fig. 4 below the horizontal dashed line and on the left of the vertical dashed line. There are 445 such solutions in total. From a global perspective, all these solutions are strictly superior to the real-world CU landscape as they feature both a higher average integration score and a lower average dissimilarity score.

At the same time, it is possible that some individual countries will prefer the real-world CU landscape over some of these solutions, because they fare better as part of their current real-world CU (at the expense of other countries). This feature is taken into account in the next subsection.

#### Customs Union Perspective

**Fig. 5:**
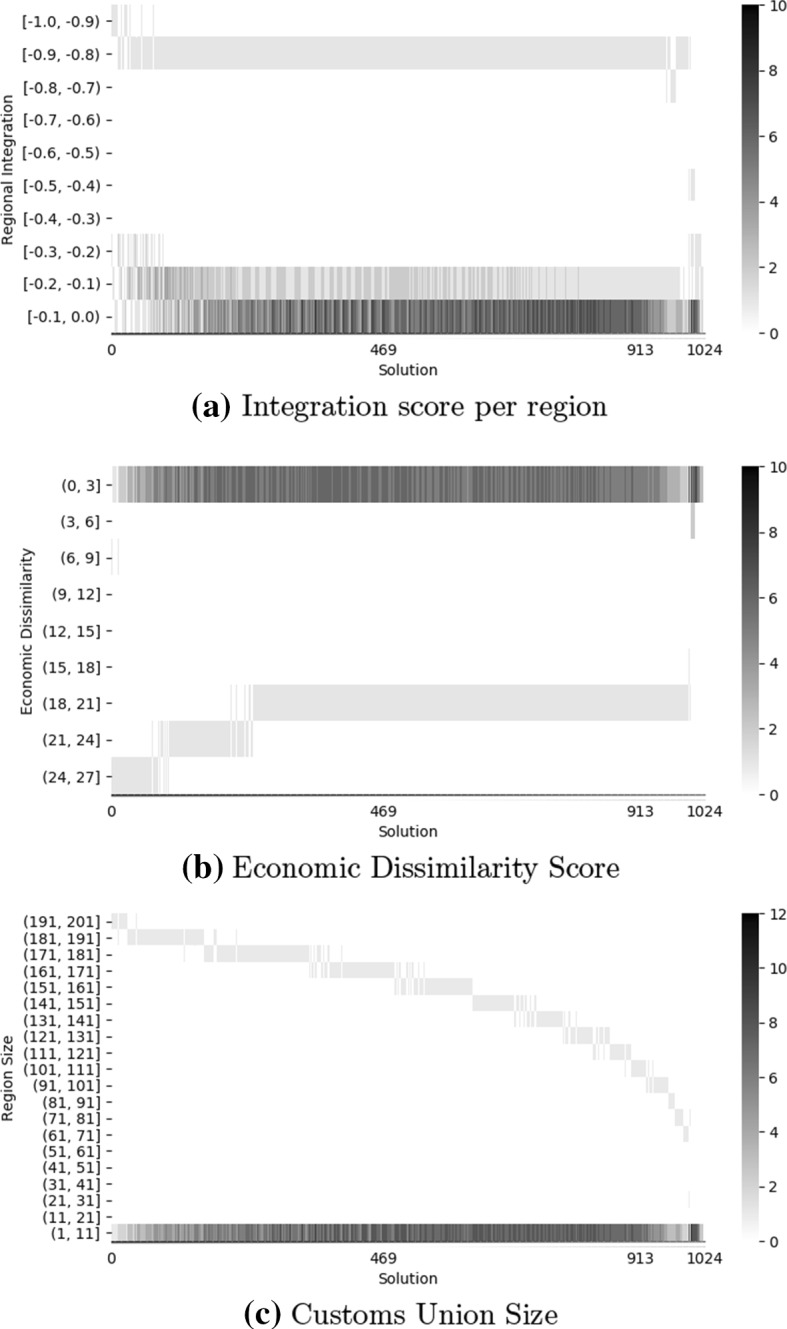
Heatmaps depicting characteristics of the regions gathered in bins in each non-dominated solution found by NSGA-II (with solutions sorted based on an increasing mean regional integration). Regions of the same solution are shown on the same x-axis. The solutions 469 and 913 delimit the solutions that dominate the configuration of real-world customs unions (i.e., solutions that perform better, on average, on both objectives)

We now evaluate the characteristics of each region (i.e., CU) in every non-dominated solution (as identified in Fig. 4, with solutions sorted based on an increasing mean regional integration, i.e., in the order they appear in from left to right). The results are illustrated in Fig. 5 which shows heatmaps of the integration score (Panel a), dissimilarity score (Panel b), and size (Panel c) of the CUs in every non-dominated solution found by the algorithm. In contrast, characteristics of the real world CUs are listed in Table 2.

**Table 2:** Characteristics of CUs in the real world

Customs Union	Integration score	Economic dissimilarity score	CU size
Southern Common Market (MERCOSUR)	− 0.1392	5.7509	4
Central American Common Market (CACM)	− 0.1583	6.3292	6
Caribbean Community and Common Market (CARICOM)	− 0.0929	15.5787	14
Economic Community of West African States (ECOWAS)	− 0.0891	15.5241	15
Gulf Cooperation Council (GCC)	− 0.1100	4.6543	6
Economic and Monetary Community of Central Africa (CEMAC)	− 0.0491	15.1815	6
East African Community (EAC)	− 0.0930	5.2738	5
Andean Community (CAN)	− 0.0762	2.7406	4
Eurasian Economic Union (EAEU)	− 0.1353	4.7694	5
Southern African Customs Union (SACU)	− 0.1342	6.4368	5
European Community (EC)	− 0.6447	16.6562	28

While the Table 2, we see that the real world contains several CUs with economic dissimilarity scores between 2 and 17, and sizes between 4 and 28 countries. In contrast, Fig. 5 shows that most of the found non-dominated solutions have a different composition, featuring one large CU (more than 100 countries), several small CUs (less than 5 countries), and almost no medium size CUs. In particular, this applies to the found solutions that dominate the real-world composition of CUs in both objectives (i.e., the solutions between 469 and 913). This is an interesting finding as it suggests that the non-dominated solutions favour the creation of large CUs while leaving many other countries out. Note however, that since our algorithm has no guaranty of optimility, it might be possible that it is missing (not finding) non-dominated solutions with average size CUs.

For example, solution 469 (corresponding to the first solution below the horizontal reference line in Fig. 4) consists of one large CU comprising 168 countries and several smaller CUs with less than 2 countries each. The mean regional integration score of solution 469 is − 0.0539 and the mean economic dissimilarity score is 0.8670. The respective values for the real-world composition of CUs are − 0.0152 and 0.8751. Thus, solution 469 dominates the real-world composition of CUs in both objectives.

**Fig. 6:**
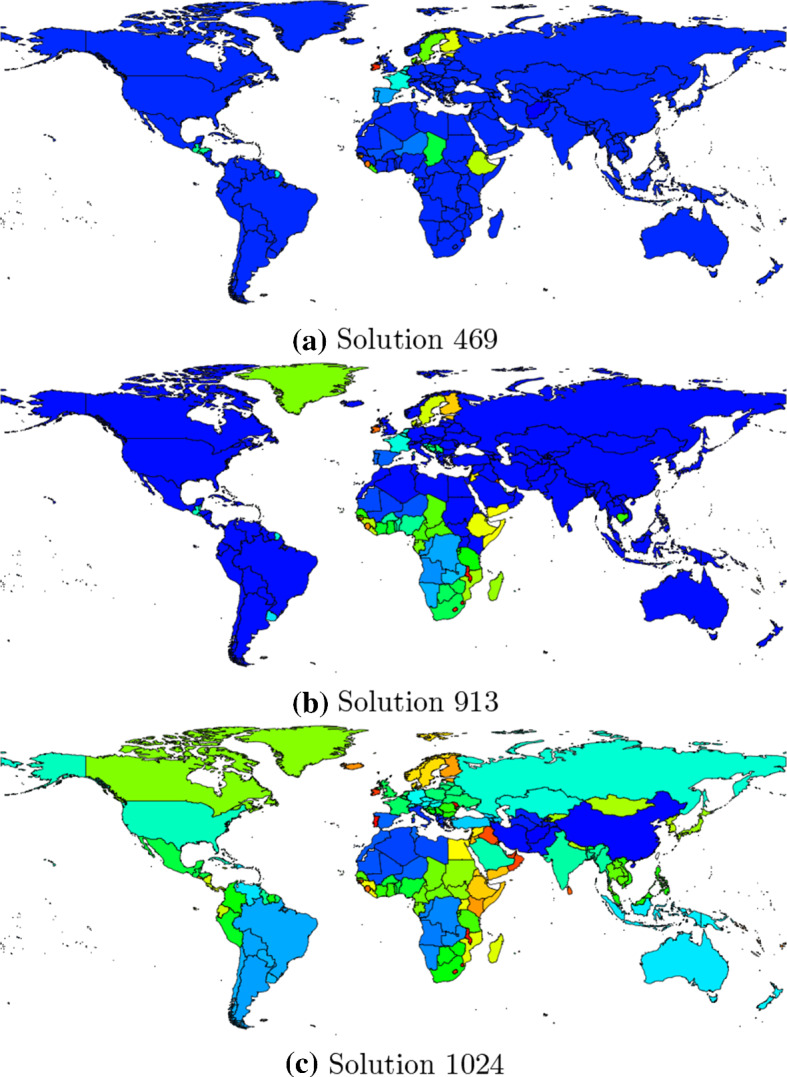
Composition of selected non-dominated solution found by NSGA-II. Bordering countries of the same color form a region

Figure 6 graphically depicts the composition of some selected solutions found by the algorithm. Bordering countries of the same color form a region. The composition of solution 469 is shown in Panel a. Panel b shows the composition of solution 913 which corresponds to the first solution left of the vertical reference line in Fig. 4. Panel c in Fig. 6 depicts solution 1024 which features the best (i.e., smallest) value on the mean economic dissimilarity score among all non-dominated solutions found by the algorithm. At the same time, the mean regional integration score of solution 1024 is higher than the one of the real-world composition of CUs (solution 1024 is thus not strictly dominating the real-world composition of CUs, which is also indicated by the fact that it is located outside the two vertical reference lines in Fig. 5).

#### Country Perspective

**Fig. 7:**
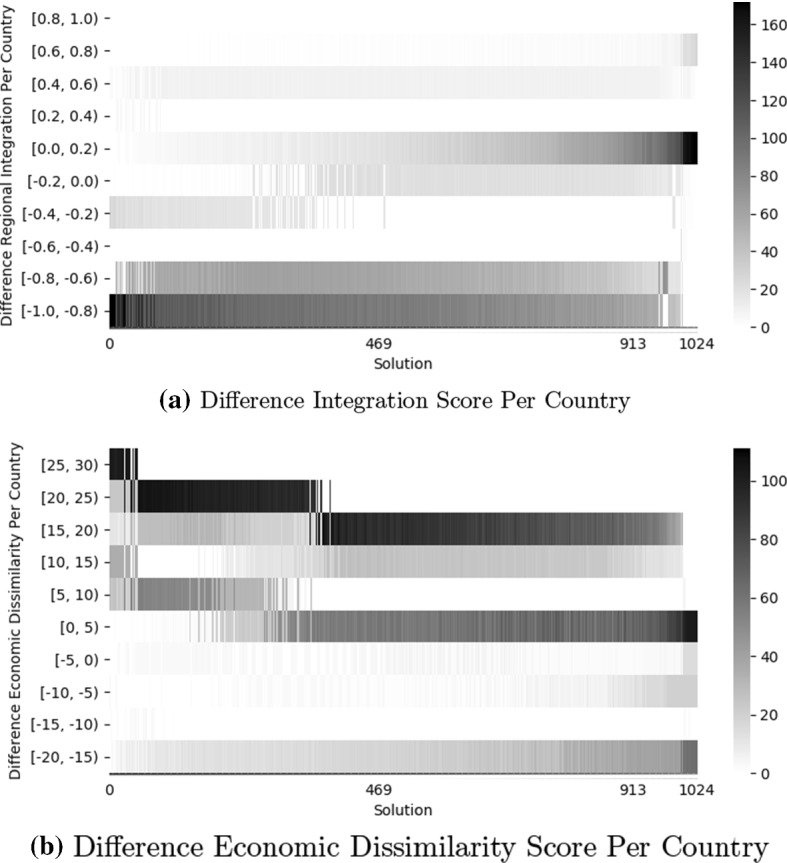
Heatmaps depicting the difference in integration score and economic dissimilarity score (in bins) for every country in each non-dominated solution found by NSGA-II (with solutions sorted based on an increasing mean regional integration) in comparison to the real world (negative values mean improved scores). Regions of the same solution are shown on the same x-axis. The solutions 469 and 913 delimit the solutions that dominate the configuration of real-world customs unions (i.e., solutions that perform better, on average, on both objectives)

Figure 7 shows heatmaps depicting the difference in integration score and economic dissimilarity of each country between every non-dominated solution (as identified in Fig. 4) and the real world.

We see from Fig. 7 that, on average, countries would experience an improvement in their regional integration scores if moved to most of the non-dominated solutions. However, the results also show that there are very few non-dominated solutions that improve the integration score of all countries (only the two non-dominated solutions on the extreme left).

From a dissimilarity point of view, we see that some countries are required to lose in terms of dissimilarity score when moving to the non-dominated solutions–even in non-dominated solutions that achieve a better average economic dissimilarity score than the real world.

**Fig. 8:**
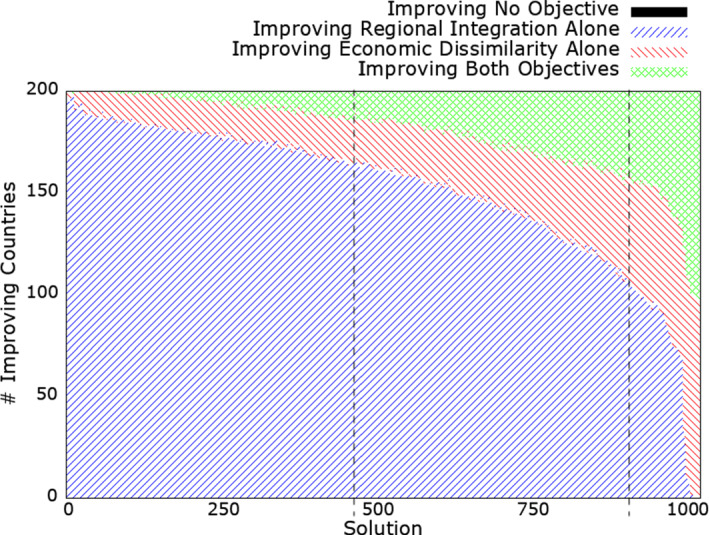
Number of countries not improving any objective, improving their regional integration alone, economic dissimilarity alone, or improving both objectives at the same time, in comparison to the customs unions in the real world in each non-dominated solution found by NSGA-II. The two dashed vertical reference lines delimit solutions that dominate the configuration of real-world customs unions (i.e., solutions that perform better, on average, on both objectives). (Color figure online)

Figure 8 shows the number of countries either not improving any objective (in black), improving their regional integration alone (in blue), improving their economic dissimilarity alone (in red), or improving both objectives at the same time (in green) for each non-dominated solution found by NSGA-II in comparison to the country’s performance when considering the CUs in the real world.

We see from Fig. 8 that, in all of the found non-dominated solutions, there are no countries in a worse situation on both objectives. Therefore, all countries will have at least some incentive to adopt one of the non-dominated solutions as this will offer them an improvement in at least one objective.

However, we also see that none of the found non-dominated solutions enables all countries to improve both objectives at the same time–irrespective of whether the non-dominated solution strictly dominates the real world on average (i.e., solutions between the two dashed vertical reference lines) or not. Therefore, there is always the possibility that some countries will object to adopting a given solution as it will worsen one of their objectives (even if it means improving the other objective). Among the solutions that strictly dominate the real world, the best solution allows up to 45 countries to improve both objectives at the same time, whereas the best solution overall allows up to 102 countries to improve their two objectives at the same time.

Moreover, we see from Fig. 8 that the number of countries improving their regional integration score alone is decreasing with the solution number (remember that solutions are sorted based on their mean regional integration score). The number of countries improving at least their regional integration score (and possibly both objectives) for the solutions dominating the real-world CU landscape ranges between 150 (75%) and 179 (89.5%). The number of countries improving at least their economic dissimilarity score (and possibly both objectives) for the solutions dominating the real world ranges between 39 (19.5%) and 95 (47.5%).

## Conclusion

This paper proposes a new mathematical approach to model the optimal size and composition of customs unions in the form of a bi-objective combinatorial non-linear problem. We also use a multi-objective evolutionary algorithm (NSGA-II) to search for the best (non-dominated) configurations based on the trade flows and economic structure of each country, which enables us to find 445 different configurations that are strictly preferable, from a global perspective, to the real-world landscape of customs unions.

Our analysis shows that the found non-dominated configurations tend to favour the creation of a few large customs unions and several gravitating smaller ones. Also, despite improving the world’s trade while limiting the economic dissimilarity, on average, the found non-dominated configurations do not improve the outcomes for all countries.

Overall, the analysis suggests that, from a global perspective, there is scope for improving the composition of existing customs unions. One possible reason that may hinder moving towards these improvements is that such changes would entail both winners and losers, as our algorithm was unable to find an alternative solution where all countries would be able to improve along both objectives. Some countries may thus have incentives to block modifications to the current customs unions that would benefit the world as a whole, but be harmful to (some of) the objectives of individual countries.

The analysis presented in this paper focuses on a static approach based on recent data. Future research may build on this approach to also evaluate the evolution of non-dominated regional configurations over time, as well as incorporating structural difference indicators at more disaggregated industry levels.

## Data Availability

The authors confirm that all data used in this study are openly available from the following sources: $\bullet$ The data on trade flows come from the Direction of Trade Statistics (DOTS) database of the International Monetary Fund (IMF). $\bullet$ The data on the sectoral composition of GDP come from the World Bank’s World Development Indicator database.

## Footnotes

1 It should be pointed out, however, that in Kenen’s logic, a distinction is made between similar/dissimilar economic structure (between economies), on the one hand, and diversified/non-diversified structure (for individual economies), on the other (Kenen, 1969). OCAs could be formed between similar non-diversified economies or between (not necessarily similar) diversified economies.

2 The enabling clause refers to the Decision by GATT signatories on Differential and More Favorable Treatment, Reciprocity and Fuller Participation of Developing Countries of 1979 which allowed certain derogations to the most-favored nation (MFN) principle in favor of developing countries. More specifically, for the purposes of our paper, paragraph 2(c) of this Decision enables developing countries to enter into preferential tariff arrangements on goods trade among themselves “[…] in accordance with criteria or conditions which may be prescribed by the Contracting Parties”. The WTO refers to such agreements, which cover only certain product categories, as partial scope agreements. CUs signed under this clause contrast with the CUs signed under paragraph 8(a) of Article XXIV of GATT 1994 as the latter refer to the elimination of duties and other restrictive regulations “with respect to substantially all the trade between the constituent territories of the union or at least with respect to substantially all the trade in products originating in such territories”.

3 In the application below there will be 485 such variables, corresponding to the real-world borders across 200 countries in our sample.

4 The dataset, algorithms, and other data required for the computation of the multi-objective metrics are available at https://github.com/takfarinassaber/OptiCUs.

5 Pareto front: a set of non-dominated solutions (i.e., better than all other solutions in one or more objectives).

6 The source code of our algorithm is publicly available at https://github.com/takfarinassaber/OptiCUs.

7 Reference front: the set of all optimal non-dominated solutions.
